# Characterizing antibody responses to mosquito salivary antigens of the Southeast Asian vectors of malaria and dengue with a human challenge model of controlled exposure: a protocol

**DOI:** 10.12688/wellcomeopenres.19049.1

**Published:** 2023-03-23

**Authors:** Sunisa Sawasdichai, Victor Chaumeau, Ellen Kearney, Praphan Wasisakun, Julie A. Simpson, David J. Price, Sadudee Chotirat, Laurent Rénia, Elke Bergmann-Leitner, Freya Fowkes, François Nosten

**Affiliations:** 1 Shoklo Malaria Research Unit, Mahidol-Oxford Research Unit, Faculty of Tropical Medicine, Mahidol University, Mae Ramat, Tak, 63140, Thailand; 2 Centre for Tropical Medicine and Global Health, Nuffield Department of Medicine, University of Oxford, Oxford, England, OX3 7BN, UK; 3 McFarlane Burnet Institute of Medical Research and Public Health, Melbourne, Victoria, VIC 3004, Australia; 4 Centre for Epidemiology and Biostatistics, Melbourne School of Population and Global Health, The University of Melbourne, Melbourne, Victoria, VIC 3010, Australia; 5 Department of Infectious Diseases, University of Melbourne, at the Peter Doherty Institute for Infection and Immunity, Melbourne, Victoria, VIC 3000, Australia; 6 Malaria Vivax Research Unit, Faculty of Tropical medicine, Mahidol University, Bangkok, 10400, Thailand; 7 A*STAR Infectious Diseases Labs, Agency for Science, Technology, and Research, Singapore, 138648, Singapore; 8 Lee Kong Chian School of Medicine, Nanyang Technological University, Singapore, 308232, Singapore; 9 The Walter Reed Army Institute of Research, Silver Spring, Maryland, MD 20910, USA; 10 Department of Epidemiology and Preventive Medicine, Monash University, Melbourne, Victoria, VIC 3052, Australia

**Keywords:** malaria, dengue, Southeast Asia, Anopheles, Aedes, exposure, saliva, clinical trial

## Abstract

**Background:** Measurement of antibody titers directed against mosquito salivary antigens in blood samples has been proposed as an outcome measure to assess human exposure to vector bites. However, only a handful of antigens have been identified and the specificity and longitudinal dynamics of antibody responses are not well known. We report the protocol of a clinical trial of controlled exposure to mosquito bites that aims to identify and validate biomarkers of exposure to bites of mosquito vector species that transmit malaria and dengue in Southeast Asia and some other parts of the world.

**Methods:** This study is an exploratory factorial randomized control trial of controlled exposure to mosquito bites with 10 arms corresponding to different species ( *Aedes aegypt*, *Ae. albopictus*, *Anopheles dirus*, *An. maculatus* and *An. minimus*) and numbers of bites (35 or 305 bites in total over 6 weeks). Blood samples will be collected from study participants before, during and after mosquito biting challenges. Candidate peptides will be identified from published literature with antigen prediction algorithms using mosquito DNA sequence data and with immunoblotting assays carried out using protein extracts of dissected mosquito salivary glands and participants samples. Antibody titers against candidate peptides will be determined in participants samples with high-throughput cutting-edge immuno-assays. Quantification of the antibody response profile over time (including an estimate of the decay rate) and the effect of the number of bites on the antibody response will be determined using linear and logistic mixed-effects models for the continuous and the binary response, respectively.

**Conclusion:** This research is expected to generate important knowledge for vector sero-surveillance and evaluation of vector-control interventions against malaria and dengue in the Greater Mekong Subregion.

**Registration:** This study is registered with clinicaltrials.gov (NCT04478370) on July 20 ^th^, 2020.

## Introduction

### Background and rationale

Mosquito-borne diseases cause significant burden in populations exposed to bites of vector species across Southeast Asia ^1^. Malaria and dengue are endemic and pose the greatest challenges. Malaria is transmitted in rural areas and multi-drug resistant falciparum malaria has been identified as a major threat to public health ^2^. Consequently, considerable investment has been made to eliminate *Plasmodium falciparum* in the Greater Mekong Subregion, the epicenter of antimalarial drug resistance ^3^. In this area, the main vectors are *Anopheles dirus*, *An. maculatus* and *An. minimus*; several other species also contribute to the transmission ^4^. The efficacy of conventional vector-control measures is low ^5– 7^ because of the ecology and biology of relevant vector species ^8– 10^, and is particularly difficult to evaluate due to the complex transmission dynamics and low rates of disease incidence ^8^. Dengue viruses are transmitted by aedine mosquitoes; the main vectors are *Aedes aegypti* and *Ae. albopictus* ^11^. Infection can cause death and overall disease burden has drastically increased over the past decades ^12^. As there is no specific treatment for dengue, prevention of infection is critical to reduce morbidity and mortality. Integrated management strategies and active involvement of homeowners is necessary to control *Aedes* mosquitoes in urban and semiurban environments. In rural areas where vector breeding sites also include a variety of natural water bodies (rock pools, tree holes and bamboo stumps), control is particularly challenging and personal protection with long-sleeve clothes and skin repellents is often the only option available ^13^.

Exposure to mosquito bites is a key parameter of the vectorial capacity equation ^14^ and its assessment is extremely informative for disease surveillance and trials of vector-control interventions. Exposure results from a combination of parameters including mosquito population density, aggressivity to humans, people movements and sleeping habits, and personal protection conferred by vector-control interventions ^15^. It is currently not possible to measure exposure accurately; mosquito biting-rate estimates are sometimes combined with data on human behaviors and vector-control to produce elusive estimates, but the cost and challenges associated with data collection are often prohibitive ^15^.

When blood feeding, mosquitoes inject saliva into the skin of vertebrates ^16^. Mosquito saliva is composed of hundreds of biologically active molecules that play essential roles in the physiology of blood feeding ^17^. Many saliva components have immunogenic properties and some of these antigens elicit detectable levels of antibody responses in the blood following biting exposure ^18^. Assessment of antibody responses directed against mosquito salivary antigens as a surrogate measure of human exposure to mosquito bites has been proposed ^19^. Individuals repeatedly bitten by mosquitoes develop a long-lasting, broad and variable sero-reactivity to mosquito salivary antigens of the biting species ^20^. Serum can cross-react with salivary antigens of other mosquito species to which an individual has never been exposed to ^21^. As a result, it can be difficult to identify antigenic peptides that elicit transient antibody responses with adequate sensitivity and specificity. Only two *An. gambiae* (gSG6-P1 and cE5) and two *Ae. sp.* (Nterm-34kDa and D7) peptides have been used for assessing exposure in large-scale epidemiological surveys and trials of vector-control ^22, 23^. However, critical parameters including sensitivity, specificity and half-life of the antibody responses have not been assessed precisely: only one prospective study strived to characterize the immune responses of humans (n = 1 subject exposed to *Culex quinquefasciatus* bites) and rabbits (n = 1 subject exposed to *Ae. aegypti* bites) to mosquito salivary antigens in a challenge model of controlled exposure ^18^. We therefore propose to conduct a worldfirst clinical trial of controlled exposure to bites of uninfected laboratory-reared *Anopheles* and *Aedes* mosquitoes to identify and validate biomarkers of exposure to dengue and malaria vector bites.

### Objectives

The primary objective of this study is to identify and validate biomarkers of exposure to bites of *An. dirus*, *An. maculatus*, *An. minimus*, *Ae. aegypti* and *Ae. albopictus*. The secondary objectives are to characterize the dose-response relationship between the number of mosquito bites and antibody titers, and to compare the performance of capillary blood spotted on filter paper and serum from venous blood for measuring antibody responses to mosquito salivary antigens.

### Trial design

This study is an exploratory factorial randomized controlled trial of controlled exposure to mosquito bites with 10 arms corresponding to different species ( *An. minimus*, *An. maculatus*, *An. dirus*, *Ae. aegypti* and *Ae. albopictus*) and numbers of bites (35 or 305 bites in total over 6 weeks). Participants will be assigned randomly to one of the 10 study arms with a 1:1 ratio using a block randomization schedule. Those with incomplete follow-up will be replaced until complete follow-up of 15 participants per arm is achieved. Serum from venous blood and capillary blood spotted on filter paper will be collected weekly from each study participant before, during and after the challenges. In addition to those described in the published literature, candidate peptides will be identified with antigen prediction algorithms using mosquito DNA sequences data (either deposited in open access databases ^24^ or generated using wild-caught mosquito specimens) and immunoproteomic assays carried out using protein extracts of dissected mosquito salivary glands and participants samples ^25^. Antibody titers will be determined with high-throughput ELISA ^26^ and mesoscale screening assays ^27^.

## Methods

### Participants, intervention and outcomes

***Study setting*.** The study will be conducted at the research center of the Shoklo Malaria Research Unit in Mae Sot, Thailand. Critical to the trial design, Mae sot is a small town in Thailand where *Anopheles* mosquitoes have disappeared after decades of development and urbanization ^28^. Therefore, people who live in Mae Sot are not exposed to *Anopheles* bites if they do not travel in rural areas located outside the city.

***Eligibility criteria*.** Volunteer eligibility to the study will be assessed using the criteria presented in Table 1.

**Table 1.** Eligibility criteria.

Inclusion criteria
Generally healthy male or female aged 18 to 60 years old as assessed by a medical doctor
Thai, Burmese or Karen ethnicity
Living in Mae Sot city for the last 12 months
Able to tolerate direct mosquito exposure
Exclusion criteria
History of travel in a rural area ( *i.e.*, where participant may be exposed *Anopheles* bites) in the last 12 months, or plan to do so during the study
Medication or condition deemed to interfere with the outcome measure or increase the risk of an adverse reaction to the study procedures (hypersensitivity to mosquito bites, atopy, systemic mastocytosis, immunodeficiencies, Epstein-Barr virus-associated lymphoproliferative disease, and longue-course oral treatment with a steroidal anti-inflammatory drug)
Moderate and severe anaemia (haemoglobin concentration less than 110 g/L of blood)
Pregnancy
Breastfeeding

***Intervention*.** Participants will be exposed to bites of laboratory-adapted colonies of *An. minimus*, *An. maculatus*, *An. dirus*, *Ae. aegypti* or *Ae. albopictus* weekly for six weeks (seven challenges per participant in total). Participants in the low-exposure arms will be challenged on each occasion with five mosquito bites (35 bites in total), those in the high-exposure arms will be challenged once with five bites and then six times with 50 bites (305 bites in total). In order to assess participant skin reactions to mosquito bites, three bites will be administered on one participant arm using single mosquitoes in 50 mL tubes topped with netting material. The remaining mosquitoes will be put into plastic cups of 10 cm in diameter topped with netting material and offered to feed on participant counter arm, calf, thigh or back skin. The mosquitoes will be left undisturbed and allowed to feed for 30 min. The number of bites actually received by the participant will be assessed by counting the number of engorged mosquitoes at the end of the exposure time. If not all mosquitoes engorge, the participant will be exposed to additional mosquitoes using the same procedure in order to reach the target number of bites. All challenges will be performed with 5- to 7-day-old starved nulliparous female imagoes ( *i.e.*, that have never blood fed before) of laboratory-adapted mosquito colonies reared in insectaries. Participants with hypersensitivity to mosquito bites will be withdrawn from the study. In order to increase adherence to the study protocol, the study coordinator will call participants on the day before a scheduled visit and remind them about the appointment. Participants who miss a visit will be given the opportunity to come for a retake until the day of the next scheduled visit. All participants will be provided antipruritic medication (chlorphenoxamine cream and cetirizine pills) to relieve itching from mosquito bites. Participants will be informed during the screening visit about the importance of avoiding being bitten by *Anopheles* mosquitoes during the study and how to do so. They will be provided with an insecticide-impregnated mosquito bed net (PermaNet 2.0®, Vestergaard) and skin repellent (N,N-diethyl-meta-toluamide, D.E.E.T., 20%). They will be asked not to travel to rural areas and the travel history will be recorded at every visit.

***Outcomes*.** The primary outcome of this study will be the participant humoral immune responses directed against mosquito salivary antigens. The measurement variables will be the amino-acid sequence of candidate peptides, and the titers of antibodies directed against these peptides determined in participants blood samples (serum from venous blood and capillary blood spotted on filter paper) collected at one-week intervals before, during and after the challenges. Antibody titers, assessed by measuring the optical density of immunoassay reactions, will be analyzed as a continuous outcome and as a binary outcome (seropositive/seronegative compared to unexposed sera). The size and type of participant skin reactions to mosquito bites will be assessed after every challenge. Reactions greater than 30 mm in diameter, ecchymosis, vesicle, blister, bullae, Skeeter syndrome and systemic symptoms (generalized urticaria, angioedema and anaphylaxis) will require withdrawing the participant from the study.

***Participant timeline*.** The participant timeline is presented in Table 2. Volunteers interested in participating in the study will be appointed for enrollment and the eligibility of those who consent will be assessed. Eligible participants will be appointed to attend weekly visits for 16 weeks and the complete follow-up will be 112 days. Participants will be challenged with mosquito bites seven times between day 14 and day 56. Immediate skin reactions will be recorded 20 to 30 minutes after every challenge and delayed skin reactions will be recorded 24 to 36 hours after the first and second challenges. The level of antibody titers against mosquito salivary antigens will be measured in participant serum from venous blood and capillary blood spotted on filter paper collected at one-week intervals for the entire follow-up.

**Table 2.** Participant timeline.

Study period																				
	Enrollment	Allocation	Post-allocation																	Closeout
Visit	1	2	3	4	5	6	7	8	9	10	11	12	13	14	15	16	17	18	19	20
Time point (in days)	-30 to 0	0	7	14	15	21	22	28	35	42	49	56	63	70	77	84	91	98	105	112
Enrollment:																				
Eligibility screen	x																			
Informed consent	x																			
Allocation		x																		
Intervention:																				
Challenge				x		x		x	x	x	x	x								
Assesments:																				
Antibody titers																				
Skin reactions				x	x	x	x	x	x	x	x	x	x	x	x	x	x	x	x	x
Physical examination	x	x	x	x		x		x	x	x	x	x								
Complete blood count	x																			
Pregnancy and lactation	x																			
Vital signs	x	x	x	x		x		x	x	x	x	x	x	x	x	x	x	x	x	x
Medical history	x																			
Travel history	x	x	x	x		x		x	x	x	x	x	x	x	x	x	x	x	x	x
Concomitant medication	x	x	x	x		x		x	x	x	x	x	x	x	x	x	x	x	x	x
Adverse events	x	x	x	x	x	x	x	x	x	x	x	x	x	x	x	x	x	x	x	x

***Sample size*.** There is no data to calculate the sample size *a priori* because the characteristics of immune responses to candidate peptides is not known at this stage. A sample size of 15 participants with complete follow-up per study arm was deemed appropriate for this study given the number of repeated assessments and expected variation in the continuous individual antibody responses. Participants with incomplete follow-up will be replaced to ensure there are 15 participants per study arm with complete followup.

***Recruitment*.** Information on the study will be spread through word of mouth by the study team to people who live in Mae Sot city and people interested in participating will be invited to contact the study team. In order to reach the planned sample size, volunteers interested to take part in the study will be asked to spread information to their network of acquaintances.

### Assignment of intervention

***Allocation sequence generation*.** A block randomization schedule will be generated using the *block.random* function of the R package *psych* version 1.8.12 ^29^ with variables species ( *An. minimus*, *An. maculatus*, *An. dirus*, *Ae. aegypti* and *Ae. albopictus*) and dose (35 or 305 bites in total), yielding an ordered list of 15 blocks with 10 participants per block randomly assigned to one of the 10 study arms.

***Allocation concealment mechanism*.** An allocation sequence will be implemented using individual, sealed and sequentially numbered envelopes. Following screening and eligibility assessment, participants will be assigned to a study arm during visit two using the randomization schedule.

***Implementation*.** An allocation sequence will be generated by a study investigator. At the beginning of the study, the study coordinator will prepare a set of case report forms (CRFs) with preprinted subject identification codes and attach the sealed envelope containing intervention allocation to the CRF. Study nurses will then assign a subject identification code to participants by chronological order of enrollment in the study and the envelope will be opened during visit two.

***Blinding*.** Allocation of intervention will be masked to outcome assessors (laboratory personnel who will process the serum samples) and data analysts. In order to do so, allocation of intervention in study datasets that contain this information will be masked until the results of the final analysis are made available to study investigators.

### Data collection, management and analysis

***Data collection methods*.** In addition to biomarkers of exposure reported in the literature ^22^, candidate peptides to be included in the test panel will be identified with antigen prediction algorithms ^30^ using DNA sequences data (either publicly available or generated from wild-caught mosquito specimens) and with immunoproteomic assays ^25^ using protein extract of dissected mosquito salivary glands and participants samples collected during visits four, 18 and 20. Antibody titers will be determined with established high-throughput ELISA ^26^ and mesoscale screening assay ^27^. Antibody titers, assessed by measuring the optical density of immunoassay reactions, will be analyzed as a continuous outcome measure and used to define a binary responder status. Optical density measurements in test samples will be normalized over a blank reaction carried out with reaction buffer instead of the test sample in order to subtract noise from the signal. The seropositivity threshold of the assay will be set using reference serum specimens of individuals not exposed to bites of anopheline or aedine mosquitoes and defined as three standard deviations above the mean optical density measured in this unexposed population. Reactions with an optical density above this cut-off will be considered seropositive ( *i.e.*, presence of antibodies recognizing the test peptide in participant serum sample). Participants with incomplete follow-up will be replaced to achieve a sample size of 15 participants per arm with complete follow-up, however, their data will still be included in the final analysis.

***Data management*.** Data will be managed by a dedicated data management team composed of a data manager and data entry clerks independent from the study team. The data management team will design the CRF, build the study database, capture the data and perform quality checks. All study data with the exception of the results of the complete blood count (CBC) test will be recorded on the CRF and entered in the study database. The results of the CBC test, outputted by the machine and stored electronically in a separate laboratory database, will be linked to the study database using the subject identification code (printed results will also be attached to the CRF). The study database will be built using MACRO software and stored on a secured server. Double-entry of data critical to the analysis (age and sex of participants, allocation of intervention, visit dates and actual number of bites administered during each challenge) will be carried out. Validation rules allowing range checks for data values will be incorporated into the database during its design.

***Statistical methods*.** The change in individual antibody titers (as a continuous outcome and binary response) over time will be analyzed using generalized linear mixed-effects models. Optical density measurements ( *i.e.*, antibody titers) will be analyzed using a Gaussian distribution family and identity link function ( *i.e.*, a linear mixed-effects model) and binary responder status will be analyzed using a Binomial distribution family and a logit link function ( *i.e.*, a logistic mixed-effects model). Age and sex of participant, mosquito species, number of bites and follow-up period (baseline, exposure and post-exposure) will be included as covariates in the mixed-effects models and a random effect ( *i.e.*, intercept) for participant to allow random variations of immune responses in individuals. In order to investigate if changes in antibody titers vary according to mosquito species or level of exposure, interaction terms will be fitted between time, species and number of bites, respectively. Estimates from the linear mixed-effects models will be used to calculate the half-lives of each antibody measure. Results obtained with capillary blood and serum specimens will be compared using the Bland and Altman method to assess the reliability of assessing exposure with dried blood spots and point-of-care tests. Data from participants with incomplete follow-up will be retained in the final dataset and analysis will be conducted per intention to treat.

### Monitoring

***Data monitoring*.** Monitoring of trial and safety data will be carried out by an internal data monitor and a safety review board independent of the study team and sponsor, following data and safety monitoring plans established before study initiation. Data monitoring will include checks of the investigator site file, consent forms, randomization, CRFs and study logbooks, adverse and serious adverse events, sample inventory and data entry. Checks will be carried out for each randomization block upon block closeout. Detected issues will be formally reported to the principal investigator and addressed by the study team following pre-specified timelines. The safety monitoring board will be composed of a medical doctor experienced in working with the border population, a biostatistician and a dermatologist. The safety monitoring board will meet within 7 days after the occurrence of any serious adverse event and issue a formal report of its recommendations to the trial executive committee and principal investigator, potentially leading to early stopping of the trial if deemed necessary to guarantee participant safety.

***Harms*.** The main risk associated with this trial are hypersensitivity reactions to mosquito bites, skin infections and accidental transmission of mosquito-borne pathogens. Adverse events will be documented according to the standard definitions of the Common Terminology Criteria for Adverse Events guidelines ^31^. Pre-specified adverse events include blood and lymphatic system disorders (leukocytosis and eosinophilia), general disorders and administration site conditions (chills, fatigue, fever, challenge site reaction, malaise, pain, challenge site lymphadenopathy), immune system disorders (allergic reaction, anaphylaxis), infections (papulopustular rash, rash pustular, sepsis, skin infection), injury and procedural complications (bruising, venous injury), skin and subcutaneous tissue disorders (bullous dermatitis, eczema, erythema multiforme, pain of skin, pruritus, rash acneiform, rash maculo-papular, skin induration, skin ulceration, urticaria, skin atrophy, skin hyperpigmentation, skin hypopigmentation). Adverse events will be diagnosed and graded by a medical doctor of the study team; study nurses will be trained to detect adverse events for the visits that do not include a physical examination by a medical doctor. Adverse events will be managed by the study team, who will refer participants to a secondary or tertiary care center if deemed necessary. Adverse events will be reported to the study sponsor and ethics committees following applicable regulation.

***Auditing*.** An internal and external trial audit may be conducted at any time upon request by the sponsor or a third party.

### Ethics and dissemination

***Trial registration*.** This study is registered with clinicaltrials.gov ( NCT04478370 on July 20th, 2020) ^32^. The herein article reports the protocol version 2.0 dated June 14 ^th^, 2020.

***Research ethics approval*.** The protocol, informed consent form, participant information sheet and consent form will be submitted and approved by the Oxford Tropical Research Ethics Committee, the Ethics Committee of the Faculty of Tropical Medicine, Mahidol University, the Alfred Hospital Ethics Committee, Burnet Institute and the Tak Community Advisory Board, a community-based committee assembling members of the communities in which the study will be performed ^33^.

***Protocol amendments*.** Any modifications to the protocol which may impact on the conduct of the study, potential benefit of the participant or may affect participant safety, including changes of study objectives, study design, participant population, sample sizes, study procedures, or significant administrative aspects will require a formal amendment to the protocol. Such amendment will be agreed upon by the study group, approved by the Ethics Committee prior to implementation and notified to the health authorities in accordance with local regulations.

***Consent*.** During the enrollment visit, study nurses will lead a group discussion with potential participants interested in taking part in the study and meet privately with individual volunteers. During both group and individual discussions, the participant information sheet and informed consent form will be presented to attendants in appropriate language (Thai, Karen or Burmese), detailing the exact nature of the study, what it will involve for the participant, the implications and constraints of the protocol, and any risks and benefits involved in taking part. It will be clearly stated that the participant is free to withdraw from the study at any time for any reason without prejudice to future care and with no obligation to give the reason for withdrawal. Participants will be allowed as much time as they wish to consider the information, and the opportunity to question study team members or other independent parties to decide whether they will participate. Written informed consent will be obtained by asking the participant to sign and date the informed consent form. Illiterate participants will be asked to give a thumb print, leaving the date field blank and a literate impartial witness will be asked to sign and date the consent form.

***Confidentiality*.** The CRF will be pseudonymized using the subject identification code, date of enrolment, initials, sex and date of birth. Samples will be identified with a specimen number, subject identification code, initials and date. Other personal data (name, telephone number and address) will be recorded and stored separately in a password-protected electronic file stored on a secure server, allowing linkage of study data with participant’s details by the study coordinator and site investigator using the subject identification code.

***Access to data*.** Direct access to data will be granted to authorized representatives from the sponsor, Burnet Institute, A*STAR Infectious Diseases Labs, Walter Reed Army Institute of Research, Shoklo Malaria Research Unit, Mahidol-Oxford Research Unit, and any host institution, ethics committee and regulatory authorities for monitoring, audits and inspections of the study to ensure compliance with regulations.

***Ancillary and post-trial care*.** The project is covered under the sponsorship of the University of Oxford. The university has a specialist insurance policy in place which would operate in the event of any participant suffering harm as a result of their involvement in the research.

***Dissemination policy*.** The scientific integrity of the project requires that the data from this study be analyzed study-wide and reported as such. All presentations and publications are expected to protect the integrity of the major objectives of the study; data that break the blind will not be presented prior to the release of mainline results. Each paper, abstract or presentation will be submitted to the principal investigator and other contributors for review of its appropriateness and scientific merit prior to submission. Every attempt will be made to reduce to an absolute minimum the interval between the completion of data collection and the release of the study results. The study results will be released to the participating physicians, referring physicians, patients and the general medical community as they become available during and after the study.

### Study status

The study started on January 21 ^st^, 2021, and the last visit was on September 14 ^th^, 2022. Laboratory processing of the study samples is on-going.

## Data Availability

No data are associated with this article.

## Reporting guidelines

Zenodo: SPIRIT checklist for ’Characterizing antibody responses to mosquito salivary antigens of the Southeast Asian vectors of malaria and dengue with a human challenge model of controlled exposure: a protocol’. https://doi.org/10.5281/zenodo.7703965.
