# Supplementary material for: Characterizing antibody responses to mosquito salivary antigens of the Southeast Asian vectors of malaria and dengue with a human challenge model of controlled exposure: a protocol
Source: Wellcome Open Res. 2023 Jul 11;8:135. Originally published 2023 Mar 23. [Version 2] doi: 10.12688/wellcomeopenres.19049.2 (PMC10338987; doi:10.12688/wellcomeopenres.19049.2)
Supplement: Peer review history

# Peer review: Reviewer response for version 2

**DOI:** 10.21956/wellcomeopenres.21758.r62568

**Published:** 2023-07-12

**Authors:** Julien Pompon

**Affiliations:** 1 MIVEGEC, IRD, CNRS, Universite de Montpellier, Montpellier, Occitanie, France

The authors correctly addressed all my comments. I Approve the paper for indexing.

Is the study design appropriate for the research question?

Yes

Is the rationale for, and objectives of, the study clearly described?

Yes

Are sufficient details of the methods provided to allow replication by others?

Partly

Are the datasets clearly presented in a useable and accessible format?

Yes

Reviewer Expertise:

Flaviviral transmission, molecular entomology

I confirm that I have read this submission and believe that I have an appropriate level of expertise to confirm that it is of an acceptable scientific standard.

# Peer review: Reviewer response for version 1

**DOI:** 10.21956/wellcomeopenres.21120.r56423

**Published:** 2023-05-22

**Authors:** Paolo Gabrieli

**Affiliations:** 1 Department of Biosciences, Universita degli Studi di Milano, Milan, Lombardy, Italy

The authors in this manuscript present the protocol to study the antibody response of humans to mosquito bites. The rationale is clear and adequate, but it lacks some details.

1. The authors put major attention on malaria. Because the chosen site lacks *Anopheles *mosquito, they can use this population as naive or not exposed to *Anopheles *bites. They include an exclusion criteria about excluding people who traveled in the rural area in the last 12 months. Based on the data in literature ^1^ the antibodies levels seem to last for more than 12 months in some subjects. I would opt for a screening of the sera of volunteers before the enrolment as exclusion criteria, to minimise, as possible, subjects with detectable levels of antibodies.
2. Even if the absence is true for *Anopheles *mosquitoes, what is the situation for *Aedes *mosquitoes? I imagine that there are in the chosen country. How can the authors deal with that?
3. In the exclusion criteria, I would suggest to add also past history of malaria/dengue/Chikungunya or other mosquito-borne pathogens. Or history of undiagnosed fever with cutaneous rash.
4.  Generally, the ELISA test is not clear to me. The authors talk about "candidate peptides". Do they test the response to whole salivary gland extracts first? Do they want to use synthetic peptides of immunogenic proteins? This is central in the study, because the authors claim to identify possible marker of exposure with this study. However, synthetic peptides do not always work ^1^ and immune response is really dependant on the single individual. How many markers do they plan to test for each species? How can decide if the marker is species specific or genus specific?
5. The authors talk about antibody response. Which class of antibodies they are going to test? Total IgG, IgG1, etc?

Is the study design appropriate for the research question?

Yes

Is the rationale for, and objectives of, the study clearly described?

Yes

Are sufficient details of the methods provided to allow replication by others?

No

Are the datasets clearly presented in a useable and accessible format?

Partly

Reviewer Expertise:

Mosquito saliva markers

I confirm that I have read this submission and believe that I have an appropriate level of expertise to confirm that it is of an acceptable scientific standard, however I have significant reservations, as outlined above.

## References

[1] : Analysis in a murine model points to IgG responses against the 34k2 salivary proteins from Aedes albopictus and Aedes aegypti as novel promising candidate markers of host exposure to Aedes mosquitoes. *PLoS Negl Trop Dis* .2019;13(10) : 10.1371/journal.pntd.0007806 e0007806 10.1371/journal.pntd.0007806 31618201PMC6816578

# Response

**Published:** 2023-06-23

**Authors:** Victor Chaumeau

**Affiliations:** Shoklo Malaria Research Unit, Thailand

We thank the reviewer for his feedback on the manuscript. A point-by-point answer to reviewer’s comments is given below:

 1. The authors put major attention on malaria. Because the chosen site lacks *Anopheles* mosquito, they can use this population as naive or not exposed to *Anopheles* bites. They include an exclusion criteria about excluding people who traveled in the rural area in the last 12 months. Based on the data in literature, the antibodies levels seem to last for more than 12 months in some subjects. I would opt for a screening of the sera of volunteers before the enrollment as exclusion criteria, to minimize, as possible, subjects with detectable levels of antibodies.

**Response**: To our knowledge, the decay rate of antibodies directed against mosquito salivary proteins is not known in detail and likely varies with the nature of the immunogenic peptide and previous exposure. Natural antibody responses to mosquito salivary proteins are indeed broad, variable, long-lasting and sometime cross-react with salivary proteins of multiple mosquito species. The aim of this study is to identify biomarkers of exposure that can be used to reflect changes in transmission dynamics in people who live in endemic areas. Therefore, we will try to identify immunogenic peptides that elicit short, sensitive and specific antibody responses. Baseline reactivity to salivary proteins that generate long-lasting antibody responses is, in our opinion, not a relevant exclusion criteria for this study.

 2. Even if the absence is true for *Anopheles* mosquitoes, what is the situation for *Aedes* mosquitoes? I imagine that there are in the chosen country. How can the authors deal with that?

**Response**: Avoiding participant exposure to mosquito bites not related to the biting challenges is indeed key to the design of this study, which is focused on malaria. Unlike *Anopheles* mosquitoes, *Aedes* mosquitoes are ubiquitous and therefore it will not be possible to avoid participant exposure to *Aedes* bites. This is a limitation to the evaluation of antibody responses to *Aedes* salivary proteins which will be treated as a secondary outcome. The evaluation of antibody responses to *Aedes* salivary proteins will be descriptive and limited to before/after comparisons. This point has been clarified in the revised version of the manuscript.

**Revision to the paragraph Study setting:** “Unlike *Anopheles* mosquitoes, *Aedes* are ubiquitous and it will not be possible to avoid participant exposure to *Aedes* bites not related to the study challenges. This is a limitation to the evaluation of antibody responses to *Aedes* salivary proteins, which will be treated as a secondary outcome and analyzed descriptively.”

**Revision to the paragraph Statistical methods:** “The analysis of antibody responses to *Aedes* salivary proteins will be descriptive and limited to before-after comparisons among exposed participants because participant exposure to *Aedes* bites not related to the study challenges could not be avoided.”

 3. In the exclusion criteria, I would suggest to add also past history of malaria/dengue/Chikungunya or other mosquito-borne pathogens. Or history of undiagnosed fever with cutaneous rash.

**Response**: It is not clear to us why the reviewer suggests to exclude people with previous mosquito-borne infection from the study. To our knowledge, the effect of previous mosquito-borne infection on baseline serum reactivity to mosquito salivary proteins has not been described precisely. Excluding people with previous history of mosquito-borne infection would have been unreliable since many people have only limited access to health care and many infections are asymptomatic. Moreover, it would have created difficulties in recruiting participants in this population where dengue and malaria are endemic, and where outbreaks of Chikungunya have occurred in recent years.

 4. Generally, the ELISA test is not clear to me. The authors talk about "candidate peptides". Do they test the response to whole salivary gland extracts first? Do they want to use synthetic peptides of immunogenic proteins? This is central in the study, because the authors claim to identify possible marker of exposure with this study. However, synthetic peptides do not always work and immune response is really dependent on the single individual. How many markers do they plan to test for each species? How can decide if the marker is species specific or genus specific?

**Response**: In order to decipher antibody responses, whole gland extract, saliva, recombinant proteins and recombinant peptides will be tested in ELISA and electrochemiluminescence screens. There are several advantages and limitations associated with the use of either whole gland extract and recombinant peptides. In general, salivary gland protein extracts are impractical to use in large-scale epidemiological studies. Therefore, the ultimate goal of this study is to identify short species-specific and genus-specific recombinant peptides. Detailed information on the screening panel is not known at this stage. The number and nature of salivary proteins tested will depend on the published literature, the output of in silico B-cell epitope predictions and immunoproteomic experiments. The comparison of antibody responses in participants of the different study arms will be used to assess cross-reactivity of the antibody responses to different mosquito species or genus (see Statistical Methods: “In order to investigate if changes in antibody titers vary according to mosquito species or level of exposure, interaction terms will be fitted between time, species and number of bites, respectively.” The reviewer’s point has been clarified in the revised version of the manuscript.

**Revision to the paragraph** **Data collection methods: **In order to decipher antibody responses, tests will be carried out with salivary gland protein extracts, recombinant proteins and recombinant peptides (including orthologs in different mosquito species). However, the ultimate goal of this study is to identify short species-specific and genus-specific recombinant peptides that can be used for large-scale vector serosurveillance.

 5. The authors talk about antibody response. Which class of antibodies they are going to test? Total IgG, IgG1, etc?

**Response**: We are going to investigate different classes and subtypes of immunoglobulins including IgM, IgG1, IgG2, IgG3, IgG4 and IgA. This has now been clarified in the revised version of the manuscript.

**Revision to the paragraph** **Data collection methods: **The specific titer of several immunoglobulin isotypes and subclasses (including IgM, IgG1-4, IgA and IgE) will be measured separately.

# Peer review: Reviewer response for version 1

**DOI:** 10.21956/wellcomeopenres.21120.r56424

**Published:** 2023-05-12

**Authors:** Cassandra Modahl

**Affiliations:** 1 Centre for Snakebite Research and Interventions, Liverpool Institute of Tropical Medicine, Liverpool, UK

The clinical trial protocol “Characterizing antibody responses to mosquito salivary antigens of the Southeast Asian vectors of malaria and dengue with a human challenge model of controlled exposure” reports on a study being conducted involving the controlled exposure of individuals to mosquito bites and the quantification of antibody responses to mosquito salivary proteins over time and in relation to the number of bites. I have the following suggestions for improvement:

**Abstract:**

 What is the high-throughput cutting-edge immuno-assay? In the Data collection, management and analysis it details a high-throughput ELISA and mesoscale screening assay (using electrochemiluminescence I assume from the cited paper). Is there a reason why both of these measurements are being taken, and which one is being referenced in the abstract?

**Intervention:**

 Are these bite numbers realistic? How many bites maximum is an average individual exposed to? Bites are also being assessed by if a mosquito has successfully blood-fed, but there could also be exposure to mosquito salivary proteins from unsuccessful blood-feeding attempts.

 Why such a focus on avoiding *Anopheles *bites when *Aedes *responses are also being evaluated?

**Recruitment:**

 If recruitment is only being done by word of mouth, will this create any bias? Will there be any considerations to making sure there are equal numbers of recruited males and females, and ages?

**Data collection, management, and analysis:**

 Could more detail be provided regarding the antigens? For the biomarkers of exposure previously reported in literature, will recombinant forms of these antigens be used? For the antigens identified using prediction algorithms, is it is just the antigen epitopes that will be synthesized and used to coat the ELISA plates? Will these be specific for each species?

 In the case of the salivary gland extracts, there will likely be a lot of non-salivary proteins present in the mixture too as there are housekeeping proteins present in salivary gland tissues that are not secreted into the saliva. These housekeeping proteins will likely share sequence conservation across the tested mosquito species, making it difficult to evaluate vector species-specific antibody responses.

Is the study design appropriate for the research question?

Partly

Is the rationale for, and objectives of, the study clearly described?

Yes

Are sufficient details of the methods provided to allow replication by others?

Partly

Are the datasets clearly presented in a useable and accessible format?

Yes

Reviewer Expertise:

Proteomics, immunology, neglected tropical diseases, bioinformatics

I confirm that I have read this submission and believe that I have an appropriate level of expertise to confirm that it is of an acceptable scientific standard, however I have significant reservations, as outlined above.

# Response

**Published:** 2023-06-23

**Authors:** Victor Chaumeau

**Affiliations:** Shoklo Malaria Research Unit, Thailand

We thank the reviewer for her feedback on the manuscript. A point-by-point answer to reviewer’s comments is given below: What is the high-throughput cutting-edge immunoassay? In the Data collection, management and analysis it details a high-throughput ELISA and mesoscale screening assay (using electrochemiluminescence I assume from the cited paper). Is there a reason why both of these measurements are being taken, and which one is being referenced in the abstract?

**Response**: The wording high-throughput cutting-edge immunoassay has been revised to ELISA and electrochemiluminescence immunoassays, and an explanation of the reason why both of these measurements are being taken is now provided.

**Revision to the paragraph Data collection methods** – “A screening panel will be constituted and the antibody titers against the candidate markers included in the panel will be determined with ELISA and electrochemiluminescence immunoassays [26, 27]. Both measurements are being taken because different investigating centers implement different technologies, and because high-throughput assays will be ultimately needed to carry out large-scale vector serosurveillance studies. Moreover, electrochemiluminescence assays can be multiplexed and the analytical performances of this technology are sometime claimed to be superior to that of ELISA [33, 34]. Are these bite numbers realistic? How many bites maximum is an average individual exposed to? Bites are also being assessed by if a mosquito has successfully blood-fed, but there could also be exposure to mosquito salivary proteins from unsuccessful blood-feeding attempts.

**Response**: Yes, the numbers of bites used in this study are realistic. These numbers are based on previous entomological investigations conducted in this area and published reports of human challenge with mosquito bites. Additional details and references to the published literature were added in the revised version of the manuscript (see our response to comment 4 from reviewer Julien Pompom). We acknowledge the reviewer’s point on the potential bias of assessing the number of bites from blood-feed mosquitoes. The methodology was chosen because it will not be possible to directly observe and record the actual number of bites received by participants during the challenges. We assumed that the bias will be distributed at random across participants and challenges. Moreover, the number of bites in the high exposure group is one order of magnitude bigger than in the low exposure group. This difference was deemed large enough to ensure that the number of bites in the low exposure group will be actually smaller than in the high exposure group, despite the random bias associated with counting blood-fed mosquitoes as a proxy of the number of bites. Why such a focus on avoiding *Anopheles* bites when *Aedes* responses are also being evaluated?

**Response**: Avoiding participant exposure to mosquito bites not related to the biting challenges is indeed key to the design of this study, which is focused on malaria. Unlike *Anopheles* mosquitoes, *Aedes* mosquitoes are ubiquitous and therefore it will not be possible to avoid participant exposure to *Aedes* bites. This is a limitation to the evaluation of antibody responses to *Aedes* salivary proteins which will be treated as a secondary outcome. The evaluation of antibody responses to *Aedes* salivary proteins will be descriptive and limited to before/after comparisons. This point has been clarified in the revised version of the manuscript.

**Revision to the paragraph Study setting:** “Unlike *Anopheles* mosquitoes, *Aedes* are ubiquitous and it will not be possible to avoid participant exposure to *Aedes* bites not related to the study challenges. This is a limitation to the evaluation of antibody responses to *Aedes* salivary proteins, which will be treated as a secondary outcome and analyzed descriptively.”

**Revision to the paragraph Statistical methods:** “The analysis of antibody responses to *Aedes* salivary proteins will be descriptive and limited to before-after comparisons among exposed participants because participant exposure to *Aedes* bites not related to the study challenges could not be avoided.”

 If recruitment is only being done by word of mouth, will this create any bias? Will there be any considerations to making sure there are equal numbers of recruited males and females, and ages?

**Response: **No, we don’t think that recruiting participants by word of mouth will be a significant source of bias in this study. Age and sex will not be taken into account in the randomization procedure because the sample size is too small and it will create difficulties in enrolling participants, but they will be included as fixed effect covariates in the models to adjust for their potential effects on outcome measures.

 Could more detail be provided regarding the antigens? For the biomarkers of exposure previously reported in literature, will recombinant forms of these antigens be used? For the antigens identified using prediction algorithms, is it is just the antigen epitopes that will be synthesized and used to coat the ELISA plates? Will these be specific for each species?

**Response**: Detailed information on the antigens is not known at this stage. In order to decipher antibody responses, tests will be carried out with salivary gland protein extracts, recombinant proteins and recombinant peptides (including orthologs in different mosquito species). However, the ultimate goal of this study is to identify short species-specific and genus-specific recombinant peptides that can be used for large-scale vector serosurveillance. More details were added in the revised version of the manuscript to address the reviewer’s point.

**Revision to the paragraph Data collection methods:** In order to decipher antibody responses, tests will be carried out with salivary gland protein extracts, recombinant proteins and recombinant peptides (including orthologs in different mosquito species). However, the ultimate goal of this study is to identify short species-specific and genus-specific recombinant peptides that can be used for large-scale vector serosurveillance.

 In the case of the salivary gland extracts, there will likely be a lot of non-salivary proteins present in the mixture too as there are housekeeping proteins present in salivary gland tissues that are not secreted into the saliva. These housekeeping proteins will likely share sequence conservation across the tested mosquito species, making it difficult to evaluate vector species-specific antibody responses.

**Response**: We acknowledge the reviewer’s point about the limitations of using salivary gland protein extracts to assess exposure to mosquito bites and mosquito saliva may be used as an alternative. There are other limitations which make salivary gland and saliva extracts impractical to use for large-scale studies. Therefore, the ultimate goal of this study is to identify short species-specific and genus-specific recombinant peptides.

# Peer review: Reviewer response for version 1

**DOI:** 10.21956/wellcomeopenres.21120.r55743

**Published:** 2023-04-17

**Authors:** Julien Pompon

**Affiliations:** 1 MIVEGEC, IRD, CNRS, Universite de Montpellier, Montpellier, Occitanie, France

The authors present a protocol to identify the immunogenic antigens in mosquito saliva. The objectives are sound and the protocol is clearly written and of interest to the communities studying viral transmission by mosquitoes. However, there are a few points that need to be addressed before indexing:

**In Introduction:**

1. There is no mention of the two vaccines against DENV that have been licenced. Please mention them and explain why the uptake among patients is limited.

**In Assignment of intervention:**

1. There is a confusion between 15 study arms with 10 participants in each. It is the opposite…

**General points:**

1. The participants will be selected from an urban area where they should not have been bitten by *Anopheles*. The absence of *Anopheles *bite based on geographic restriction is not very strong and there should be another way to control that the participants did not experience *Anopheles *bites. The authors could test the response to *Anopheles *salivary proteins before the challenge, for instance.
2. Why 35 and 305? The authors should explain why they selected this number of bites. For instance, compare these bites with the average number of bites in this region of the world and elsewhere.
3. Recruitment will be done through word of mouth. It seems, but maybe I am wrong, that they overestimate the recruitment power of word of mouth. What about advertising in social media? They would also recruit a more diverse crowd of participants.
4. It is not clear in the study if the authors will measure the antibodies to salivary proteins from all mosquito species or just against *Anopheles *salivary proteins; please clarify in the text.
5. More details should be given about the methods to quantify the antibody response, among others the number of salivary proteins tested, their quantity, volume of serum...

Is the study design appropriate for the research question?

Yes

Is the rationale for, and objectives of, the study clearly described?

Yes

Are sufficient details of the methods provided to allow replication by others?

Partly

Are the datasets clearly presented in a useable and accessible format?

Yes

Reviewer Expertise:

Flaviviral transmission, molecular entomology

I confirm that I have read this submission and believe that I have an appropriate level of expertise to confirm that it is of an acceptable scientific standard, however I have significant reservations, as outlined above.

# Response

**Published:** 2023-06-23

**Authors:** Victor Chaumeau

**Affiliations:** Shoklo Malaria Research Unit, Thailand

We thank the reviewer for his feedback on the manuscript. A point-by-point answer to reviewer’s comments is given below:

1. There is no mention of the two vaccines against DENV that have been licensed. Please mention them and explain why the uptake among patients is limited.

**Response**: Mention of the two vaccines against DENV that have been licensed and explanation of the reasons why the uptake among patients is limited have now been added to the revised version of the manuscript.

**Revision to the paragraph Background and rationale** – “Two vaccines against dengue viruses were licensed but the uptake among patient is limited: one is indicated in children with previous infection and the other is licensed only in Europe.”

1. There is a confusion between 15 study arms with 10 participants in each. It is the opposite…

**Response**: We have reviewed the manuscript and cannot find any text specifically stating “15 study arms”. We confirm that the sentence “A block randomization schedule will be generated using the block.random function of the R package psych version 1.8.12 [32] with variables species ( *An. minimus*, *An. maculatus*, *An. dirus*, *Ae. aegypti* and *Ae. albopictus*) and dose (35 or 305 bites in total), yielding an ordered list of 15 blocks with 10 participants per block randomly assigned to one of the 10 study arms.” is correct.

1. The participants will be selected from an urban area where they should not have been bitten by *Anopheles*. The absence of *Anopheles* bite based on geographic restriction is not very strong and there should be another way to control that the participants did not experience *Anopheles* bites. The authors could test the response to *Anopheles* salivary proteins before the challenge, for instance.

**Response**: Three blood samples will be collected from each participant at baseline (before the mosquito challenges). In the analysis, baseline reactivity will be included to take into account previous exposure by including a random intercept for participant to allow the immune response at baseline to vary between individuals (see paragraph Statistical Methods).

1. Why 35 and 305? The authors should explain why they selected this number of bites. For instance, compare these bites with the average number of bites in this region of the world and elsewhere.

**Response**: The numbers of bites used in this study were chosen based on previous entomological investigations conducted in this area and published reports of human challenge with mosquito bites. Justification of the number of bites and references to the published literature were added in the revised version of the manuscript.

**Revision to the paragraph Intervention** – “The numbers of bites were chosen based on previous entomological investigations conducted in this area [8] and published reports of human challenge with mosquito bites [29, 30, 31].”

1. Recruitment will be done through word of mouth. It seems, but maybe I am wrong, that they overestimate the recruitment power of word of mouth. What about advertising in social media? They would also recruit a more diverse crowd of participants.

**Response**: Based on our experience in working with the population on the Thailand-Myanmar border, social media were deemed inappropriate for advertising the study because they reach mostly adolescent and young adults, and exclude illiterate people and people who don’t use social media (an important proportion of the study population). Word of mouth has been used to enroll study participants in many of our studies and was not associated with difficulties in recruiting.

1. It is not clear in the study if the authors will measure the antibodies to salivary proteins from all mosquito species or just against *Anopheles* salivary proteins; please clarify in the text.

**Response**: We will measure antibody responses to salivary proteins from all mosquito species. This has been clarified in the revised version of the manuscript.

**Revision to the paragraph Outcomes** – “The primary outcome of this study will be the participant antibody responses to Anopheles salivary proteins. The secondary outcome of this study will be the participant antibody responses to *Aedes *salivary proteins.”

1. More details should be given about the methods to quantify the antibody response, among others the number of salivary proteins tested, their quantity, volume of serum

**Response**: Detailed information about the methods to quantify antibody responses is not known at this stage. The number of salivary proteins tested will depend on the published literature, the output of *in silico* B-cell epitope predictions and immunoproteomic experiments. The experimental conditions in the assays (quantity of peptide, serum dilution, incubation times etc.) will require peptide-specific and laboratory-specific optimization. This was clarified in the revised version of the manuscript.

**Revision to the paragraph Data collection methods** – “A screening panel will be constituted and the antibody titers against the candidate markers included in the panel will be determined with ELISA and electrochemiluminescence immunoassays [26, 27]. Both measurements are being taken because different investigating centers implement different technologies, and because high-throughput assays will be ultimately needed to carry out large-scale vector serosurveillance studies. Moreover, electrochemiluminescence assays can be multiplexed and the analytical performances of this technology are sometime claimed to be superior to that of ELISA [33, 34]. The specific titer of several immunoglobulin isotypes and subclasses (including IgM, IgG1-4, IgA and IgE) will be measured separately. The experimental conditions in the assays (quantity of peptide, serum dilution, incubation times etc...) are not yet known since they will require peptide-specific and laboratory-specific optimization. The final composition of the screening panel is not known at this stage. In addition to biomarkers of exposure re ported in the literature, candidate markers will be identified with antigen prediction algorithms using mosquito salivary proteins sequence data and with immunoproteomic assays using protein extract of dissected mosquito salivary glands and participants samples collected during visits four, 18 and 20 [35, 25].”

# Response

**Published:** 2023-06-23

**Authors:** Victor Chaumeau

**Affiliations:** Shoklo Malaria Research Unit, Thailand

We thank the reviewer for his feedback on the manuscript. A point-by-point answer to reviewer’s comments is given below:

 1. There is no mention of the two vaccines against DENV that have been licensed. Please mention them and explain why the uptake among patients is limited.

**Response**: Mention of the two vaccines against DENV that have been licensed and explanation of the reasons why the uptake among patients is limited have now been added to the revised version of the manuscript.

**Revision to the paragraph Background and rationale** – “Two vaccines against dengue viruses were licensed but the uptake among patient is limited: one is indicated in children with previous infection and the other is licensed only in Europe.”

 2. There is a confusion between 15 study arms with 10 participants in each. It is the opposite…

**Response**: We have reviewed the manuscript and cannot find any text specifically stating “15 study arms”. We confirm that the sentence “A block randomization schedule will be generated using the block.random function of the R package psych version 1.8.12 [32] with variables species ( *An. minimus*, *An. maculatus*, *An. dirus*, *Ae. aegypti* and *Ae. albopictus*) and dose (35 or 305 bites in total), yielding an ordered list of 15 blocks with 10 participants per block randomly assigned to one of the 10 study arms.” is correct.

 3. The participants will be selected from an urban area where they should not have been bitten by *Anopheles*. The absence of *Anopheles* bite based on geographic restriction is not very strong and there should be another way to control that the participants did not experience *Anopheles* bites. The authors could test the response to *Anopheles* salivary proteins before the challenge, for instance.

**Response**: Three blood samples will be collected from each participant at baseline (before the mosquito challenges). In the analysis, baseline reactivity will be included to take into account previous exposure by including a random intercept for participant to allow the immune response at baseline to vary between individuals (see paragraph Statistical Methods).

 4. Why 35 and 305? The authors should explain why they selected this number of bites. For instance, compare these bites with the average number of bites in this region of the world and elsewhere.

**Response**: The numbers of bites used in this study were chosen based on previous entomological investigations conducted in this area and published reports of human challenge with mosquito bites. Justification of the number of bites and references to the published literature were added in the revised version of the manuscript.

**Revision to the paragraph Intervention** – “The numbers of bites were chosen based on previous entomological investigations conducted in this area [8] and published reports of human challenge with mosquito bites [29, 30, 31].”

 5. Recruitment will be done through word of mouth. It seems, but maybe I am wrong, that they overestimate the recruitment power of word of mouth. What about advertising in social media? They would also recruit a more diverse crowd of participants.

**Response**: Based on our experience in working with the population on the Thailand-Myanmar border, social media were deemed inappropriate for advertising the study because they reach mostly adolescent and young adults, and exclude illiterate people and people who don’t use social media (an important proportion of the study population). Word of mouth has been used to enroll study participants in many of our studies and was not associated with difficulties in recruiting.

 6. It is not clear in the study if the authors will measure the antibodies to salivary proteins from all mosquito species or just against *Anopheles* salivary proteins; please clarify in the text.

**Response**: We will measure antibody responses to salivary proteins from all mosquito species. This has been clarified in the revised version of the manuscript.

**Revision to the paragraph Outcomes** – “The primary outcome of this study will be the participant antibody responses to Anopheles salivary proteins. The secondary outcome of this study will be the participant antibody responses to Aedes salivary proteins.”

 7. More details should be given about the methods to quantify the antibody response, among others the number of salivary proteins tested, their quantity, volume of serum

**Response**: Detailed information about the methods to quantify antibody responses is not known at this stage. The number of salivary proteins tested will depend on the published literature, the output of *in silico* B-cell epitope predictions and immunoproteomic experiments. The experimental conditions in the assays (quantity of peptide, serum dilution, incubation times etc.) will require peptide-specific and laboratory-specific optimization. This was clarified in the revised version of the manuscript.

**Revision to the paragraph Data collection methods** – “A screening panel will be constituted and the antibody titers against the candidate markers included in the panel will be determined with ELISA and electrochemiluminescence immunoassays [26, 27]. Both measurements are being taken because different investigating centers implement different technologies, and because high-throughput assays will be ultimately needed to carry out large-scale vector serosurveillance studies. Moreover, electrochemiluminescence assays can be multiplexed and the analytical performances of this technology are sometime claimed to be superior to that of ELISA [33, 34]. The specific titer of several immunoglobulin isotypes and subclasses (including IgM, IgG1-4, IgA and IgE) will be measured separately. The experimental conditions in the assays (quantity of peptide, serum dilution, incubation times etc...) are not yet known since they will require peptide-specific and laboratory-specific optimization. The final composition of the screening panel is not known at this stage. In addition to biomarkers of exposure re ported in the literature, candidate markers will be identified with antigen prediction algorithms using mosquito salivary proteins sequence data and with immunoproteomic assays using protein extract of dissected mosquito salivary glands and participants samples collected during visits four, 18 and 20 [35, 25].”
